# How Does Reciprocal Exchange of Social Support Alleviate Individuals’ Depression in an Earthquake-Damaged Community?

**DOI:** 10.3390/ijerph18041585

**Published:** 2021-02-08

**Authors:** Arpana Pandit, Yoshinori Nakagawa

**Affiliations:** School of Economics and Management, Kochi University of Technology, Kochi 782-8502, Japan; arpana90@gmail.com

**Keywords:** social support, earthquake, mental health, reciprocity, Nepal

## Abstract

There is ample evidence to indicate the direct effects of receiving social support on mental health during and after a disaster. However, the importance of reciprocal exchanges of social support (i.e., balanced receipt and provision of social support) in maintaining the mental health status of individuals is not widely recognized. Using equity theory and reciprocity norms as a conceptual base, we distinguished two types of social support, namely, emotional support and instrumental support, and examined the effects of reciprocal exchanges of types of support on depression in survivors of an earthquake-damaged community. To collect data, in 2019, a questionnaire survey was conducted among 295 survivors of the 2015 Gorkha Earthquake in a rural village in Nepal. Our results showed that the relationship between reciprocal exchange of support and depression varied depending on the types of support. The amount of emotional support received by the individual alleviated his/her depression only when accompanied by giving emotional support. By contrast, the net amount of instrumental support given by the individual increased his/her depression. The practical implications of the study are discussed.

## 1. Introduction

Natural disasters such as earthquakes cause loss of life, damage to property, destruction of infrastructure, and a range of harmful psychological disorders, including depression, posttraumatic stress disorder, and anxiety [1,2,3]. It has been reported that 15–20 percent of survivors will experience mild or moderate psychological disorders, while 3–4 percent will suffer severe disorders, including severe depression or severe anxiety after natural disaster [4,5]. These mental health issues can significantly impact quality of life and living conditions in disaster-affected areas. It is therefore necessary to take the mental health of survivors into account following an earthquake.

Depression is the second most commonly reported psychiatric problem in disaster research [6]. A 2011 systematic review of mental health problems after the Great East Japan earthquake reported a prevalence of depression ranging from 3.0% to 43.7% following the disaster [7]. According to the 10th edition of the International Classification of Disease (ICD-10), depressive disorders can be categorized as mild, moderate, and severe episodes in relation to degree that the patient suffers from a low mood, reduced energy, and decreased activity. The individual’s capacity for enjoyment, level of interest, and degree of concentration are reduced, and marked fatigue is seen. Sleep is generally disturbed, and appetite is diminished. Self-esteem and self-confidence are reduced, and ideas of guilt and worthlessness are often present even in mild depression [8]. Depression is associated with multiple factors, including sociodemographic factors, sociocultural influences, disaster-induced economic losses, posttraumatic exposure, cognitive and physical impairment, and loss of social connection [9].

In recent decades, a range of evidence has shown that social support modifies the mental health [10,11,12], and as a result, the role of social support has attracted a great deal of attention in disaster studies. In fact, earlier studies have found that receiving social support has a positive relationship to post disaster mental health [13,14,15,16,17,18,19]. Some studies have adopted a more refined way of conceptualizing social support, distinguishing instrumental support (e.g., someone being available to offer help with issues that require physical effort or financial aid) and emotional support (e.g., someone being available to listen or offer sympathy during crisis and hardship) [9,20,21,22]. These dimensions of social support have different implications for mental health [17,23]. Some studies have identified the positive effects of receiving social support on mental health [24,25], while others have identified positive effects of providing social support [26,27,28,29].

A few studies have been conducted that focus particularly on depression and social support following disasters [1,19,30,31,32,33,34,35]. Empirical research reveals mixed findings regarding the impact of giving and receiving social support on depression. Regarding the impacts of receiving social support, some studies have found that receiving emotional support and informational support can alleviate depressive symptoms of survivors following natural disasters [30,31]. Others have found that receiving emotional support can reduce the risk of developing depressive symptoms after an earthquake [1,32]. Watanabe et al. [33] found that receiving social support from neighbors reduced depressive symptoms among displaced older survivors following 1999 Taiwan earthquake. By contrast, Hall et al. [34] demonstrated that receiving social support sometimes increases depression among disaster survivors. With regards to the impacts of providing social support, Shakespeare-Finch et al. [35] found that providing social support increases the risk of depression severity among post-flood survivors.

In spite of this accumulation of studies, however, there is still room for further research for the following reason. Earlier studies on equity theory [36,37,38] and the norms of reciprocity [39] (see the following section for details) demonstrate that establishing a reciprocal relationship with others is important for individuals’ mental health and thus strongly suggests the insufficiency of investigating the individual effects of giving or receiving social support under the assumption that they independently influence mental health (that is, assuming there is no interaction effect between giving and receiving support).

While the association of reciprocity of social support (i.e., balance receipt and provision of social support) on the mental health is a well-investigated research topic in the psychological literature of [40,41,42,43,44,45,46,47,48], little work on this has been done in the natural disaster. It is true that there are some valuable exceptions, such as Shakespeare-Finch and Green [17] and Lebowitz [49], who found that bidirectional social support (giving and receiving) enhances psychological well-being and relational satisfaction. Even if this is the case, to authors’ knowledge, the interaction effects of giving and receiving social support on depression of individuals impacted by natural disaster have never been investigated; further, the distinction between instrumental and emotional social support has also been neglected. This study was conducted to fill this gap. Pursuing an investigation of this type must be considered to be essential, due to the natures of instrumental and emotional social support. By definition, giving and receiving instrumental support has negative and positive practical value, respectively, and thus, it seems likely that the net amount of support given (rather than reciprocity) would matter more for instrumental support, and reciprocity would matter more for emotional support.

This study investigates these concerns in a village damaged by the 2015 earthquake in Nepal (known as the Gorkha earthquake), which killed nearly 9000 people and injured nearly 22,000, all over the country [50]. The remainder of the paper is divided as follows. Section 2 presents the theoretical framework and identifies the research questions. Section 3 describes the design details of the survey and the results of the statistical analyses of the data collected in the survey, whose objective is to answer the research questions shown in Section 4. Section 5 presents the discussion and Section 6 presents the limitations and Section 7 presents the conclusion and implications of the study.

### Aim of the Study

This study aims to assess the impact of the accumulative effect (over a few years since the occurrence of the earthquake 2015) of the reciprocal exchange of social support on depression among the survivors of an earthquake-damaged community.

## 2. Theoretical Perspective

This study is guided by equity theory [36,37,38] and the notion of the reciprocity of norms [39]. Equity theory indicates that individuals experience emotional and psychological distress when the amount of support given and received by an individual are not equal to another [36,37,38]. This implies that relationships are considered to be the most satisfying in the case of a perception of balance and equality in what each partner contributes to and receives from a particular social relationship [51]. Here, it is also seen individuals become dissatisfied in social exchange within a relationship if they feel either under-benefited (giving more than receiving) or over-benefited (receiving more than giving) [45,46,52,53].

Previous studies have shown the importance of reciprocity for the maintenance of mental health and psychological well-being in an individual. For example, Roberto & Scott [54] examined the relationship between older friendships and perceived distress within a relationship and found that individuals who perceive their relationships to be equitable express less distress with all aspects of their friendships than those who perceived their friendship not to be equitable. Rook [41] found that older widowed women who reported balance exchanges within their adult children and their friends felt less lonely. Buunk and Prins [55] found that students who enjoyed reciprocal social exchanges with their best friends had lower loneliness than those who felt under-benefited or over- benefited. Other studies have examined patterns of supportive exchanges among employed older adults and found that receiving emotional support adversely affected psychological well-being among employees when support exchanges are considered to involve over-reciprocating [45]. Prior studies have examined the association between social support and psychological well-being and found that imbalances in the ratio of support given and received are associated with poor psychological well-being [44,45]. Taken together, these findings indicate that people have a deeply rooted tendency to pursue reciprocity in interpersonal relationships and that they feel distressed if they perceive their relationships to be inequitable. Buunk and Schaufeli [56] argued that reciprocity is universal and an evolutionarily rooted psychological principle, which increased the likelihood of our ancestors’ survival in the evolutionary past. Therefore, equity or reciprocity in relationships is important for maintaining social status in the community.

Pursuing another line of study, Gouldner [39] presents a set of norms of reciprocity in social exchange. For Gouldner [39] reciprocity norms form a moral code that obliges people to reciprocate benefits or assistance in their social relationships. This assessment implies that individuals are more opposed to being over-benefited, as they are motivated to reciprocate in their social relationships by internalized moral norms [45,57,58,59]. A few studies have suggested that norms of reciprocity should be incorporated when the aspects of social support are assessed [60]. When reciprocity norms are widely recognized, the degree to which reciprocity applies to people and cultures varies [61]. Reciprocity is more likely to evolve in species with longer life spans who live in a small groups and are highly dependent upon each other for survival [56]. As Hawkes [62] found, past mutual trust is not what makes friends and neighborhood better candidates for reciprocity than strangers but the greater likelihood that they will be around tomorrow. These events help create mutual consciousness among groups, a spirit of solidarity to cope with any traumatic or stressful condition, and the capacity to embrace interdependence. Reciprocity in relationships is therefore critical to maintain both the physical as well as the psychological well-being of individuals.

These theories pursue different lines, but their mutual consistency is obvious. Nahum-Shani et al. [45], acknowledging this, have developed a framework that incorporates both theories, centering the role of social norms as a mechanism that underlies the way that the pattern of support exchange affects individual well-being. Given this state of affairs, in this paper, the authors seek to integrate equity theory and norms of reciprocity. As these theories imply the existence of interactions, we set the following research questions and confirm negative answers for both questions.

Research Questions:

In light of previous findings, we derive the following research questions:

In a community severely damaged by an earthquake,

- Question 1. Does the amount of emotional support that an individual receives decrease depression, regardless of the amount of emotional support that that person gives to others?
- Question 2. Does the amount of instrumental support that an individual receives decrease depression, regardless of the amount of instrumental support that that person gives to others?

Our expectations regarding the answers are as follows. Following Mizuno et al. [47], we create four categories according to the amount of support provided and received by a given individual. The categories are as follows: low in giving and low in receiving, group 1; low in giving and high in receiving, group 2; high in giving and low in receiving, group 3; and high in giving and high in receiving, group 4, for each type of social support. On the basis of this classification, as well as the existing theory, we expect to have the following answers for the above mentioned questions.

With regard to Question 1, equity theory states that individuals who are high or low in reciprocal exchanges have the same level of depression, whereas two other groups (low in giving & high in receiving; high in giving & low in receiving) may have higher levels of depression. However, with regards to emotional support, if social connectedness is taken into account, using equity theory and norms of reciprocity, it is predicted that individuals with a high degree of reciprocal exchange may have lower depression levels, and thus the answer is expected to be negative.

With regard to Question 2, equity theory states that individuals high in reciprocal exchanges and low in reciprocal exchanges will have same level of depression, whereas the two other groups (low in giving & high in receiving; high in giving & low in receiving) may have a higher level of depression. So, if the amount of support provided is high or low in a relationship, we check whether the interaction effect holds true for instrumental support. It is predicted that individuals with high levels of giving and low levels of receiving may have higher depression levels, and thus the answer is expected to be negative.

## 3. Materials and Methods

### 3.1. Data Collection

**Figure 1:**
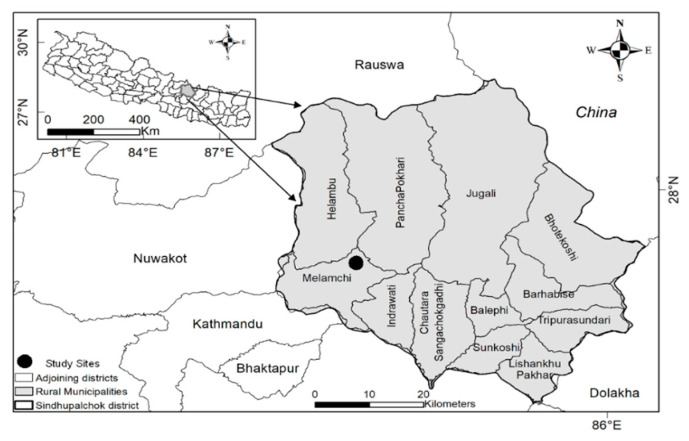
Map of Nepal Showing Study Area.

The data were collected in the municipality of Melamchi in the district of Sindhupalchowk, about 80 km northeast of Kathmandu, the capital of Nepal (Figure 1), which was one of the worst-affected districts in the 2015 earthquake. In this district, 3440 were killed, and 2101 were injured [50]. Melamchi, an administrative unit in Sindhupalchowk, consists of 13 wards. Wards 7 and 8 were chosen as the study area because: (i) they were heavily affected by the earthquake, (ii) external support and resources were limited in this area, and (iii) people depended entirely on mutual assistance after the 2015 earthquake. The 2011 census found the population, total area, and population density per square km to be 5713 people, 20.37 km^2^, and 280.46 people per km^2^, respectively [63].

A cross-sectional, face-to-face questionnaire survey was conducted from February 2019 to March 2019 among 295 subjects. The inclusion criteria were as follows: (i) 18 years of age or older, (ii) living in the current place for at least 6 months, (iii) mentally healthy to participate in the survey, (iv) head of a household, and (v) living in the village when the earthquake occurred. We used systematic random sampling to collect data satisfying all of these criteria.

Eligibility criterion (iv) entailed that the household was the primary analytical unit. This was done because households are closely bound together and cooperated in the disaster recovery. In rural Nepal in particular, it is common practice for the head of a household to be the primary spokesperson for the family. Because they have a strong sense of their household’s vulnerability to disaster [64], and are the main decision makers of their households and are well informed about their family affairs [65], this choice was supported. The survey response rate was 96%.

### 3.2. Measures

The questionnaire included the items regarding (i) demographic characteristics of the respondents (including their age, gender, education level, marital status, occupation, income, and the number of family members), (ii) earthquake exposure (damage and losses from the earthquake), (iii) mutual support activities (including the two dimensions of giving and receiving social support) and (iv) individual responses to the Patient Health Questionnaire-9 (PHQ-9).

#### 3.2.1. Earthquake Exposure

Six variables were used to assess the damage caused by the earthquake, measured with six questions: (i) injury to the respondent, (ii) injuries to respondent’s family members, (iii) loss/death of family members, (iv) damage to the house, (v) loss of food items, and (vi) loss of livestock. The respondents were requested to provide binary answers (yes or no responses) for all questions.

#### 3.2.2. 2-Way Social Support Scale

Shakespeare-Finch and Obst [22] assessed the amount of instrumental and emotional support in 2-way relationships (i.e., support that is given and received by individuals), and Bokszczanin [27] measured the amount of support provided and received in areas damaged by flood. This present study utilized 11 of 21 items regarding emotional support created by Shakespeare-Finch and Obst [22], and four of the nine items on instrumental support created by Bokszczanin [27]. Instrumental items of the former were not included because they were not appropriate for earthquake recovery and five items of the latter were deleted because they duplicated other items on emotional support and informational support. An additional 13 items were created following our field observations and an interview with a government official at the District Health Office and an official of the Nepal Red Cross Society of Sindhupalchowk. These officers had a comprehensive understanding of the damage to the district that the study site belongs to. Both of these interviews lasted for approximately 30–45 min and were conducted on 15 December 2018. It was found that the damage to be measured at the study site could not be accounted for by the two scales chosen. The emotional support items published by Shakespeare-Finch and Obst [22] were directly applicable to the earthquake recovery, but the instrumental items were not. Similarly, items on instrumental support from the Bokszczanin [27] did not cover all aspects of the damage or the support provided and received by the participants. Thus, further questions on instrumental support were developed, making 28 items in total.

Shakespeare-Finch and Obst [22] used a 4-point Likert scale, from 1 (strongly disagree) to 4 (strongly agree), and Bokszczanin [27] used a 4-point scale, from 0 (never) to 3 (many times), but in this study, participants were requested to provide binary answers, answering 1 if they had the experience of providing or receiving the social support described in the item since the earthquake and 0 otherwise. This was to avoid answers relying on individuals’ subjective feeling.

Not all of the 28 items were utilized in the statistical analysis. Specifically, to determine the set of items needed to define each of the four (2 × 2) subscales (i.e., giving vs. receiving and instrumental vs. emotional), the item-total-correlations were calculated, and items with correlations less than 0.30 were deleted [66]. This produced a final list of 19 items. (See Table 1 for details). The items not utilized in the final analysis are given in the Appendix (See Appendix A for details).

#### 3.2.3. Patient Health Questionnaire (PHQ-9)

The Patient Health Questionnaire (PHQ-9) is used to screen and diagnose depression in community settings [67]. We used the validated Nepali version of the PHQ-9 from an earlier study [68]. This tool has nine items used to record the frequency of depression symptoms over the previous 2 weeks, such as (1) little interest or pleasure in doing things and (2) feeling down depressed or hopeless. The responses were reported on a 4-point Likert scale (0 = not at all, 1 = several days, 2 = more than half of the days, and 3 = nearly every day), making the possible cumulative range from 0 to 27. Higher scores are associated with more serious depression. The two-week period here refers to the time period when the data were collected in 2019, rather than immediately after the occurrence of the earthquake in 2015. This strategy is validated from two perspectives.

First, the distress associated with the disaster may persist for a long period of time after the incident of the earthquake. Some studies have found that a bereaved family may carry a lifelong burden of depression, anxiety, and posttraumatic stress compared to the general population [69]. Furthermore, longitudinal studies after the 2011 Great East Japan Earthquake showed that posttraumatic stress decreases over time in affected areas, but depression did not [7]. Thus, it was considered important to identify factors predicting depression well after the earthquake (i.e., after the period of a few years in this study).

Second, the items presented in Table 1 include social support given or received, not only immediately after the earthquake but also over the period until 2019. Inclusion of the latter makes it reasonable to investigate the accumulative effect of social support given or received over the short and long run to the depression well in the longer run (i.e., a few years in this study).

### 3.3. Statistical Analysis

Multivariate regression analyses were conducted to describe the PHQ-9 score in terms of the four subscale scores of the 2-Way Social Support Scale, damage due to the earthquake, and sociodemographic variables. Among the 295 respondents, the questionnaires of nine were not usable, leaving the data from 286 for the analysis.

Including the four subscales of the 2-Way Social Support Scale that were highly correlated with one another (correlation coefficients for the six pairs of four items ranged between 0.13 and 0.48), the present study followed Mizuno et al. [47], who examined the relationship between the reciprocity of social support and psychological distress among Japanese older adults. (Note that, unlike the present study, Mizuno et al. [47] did not distinguish between types of social support.) Specifically, for both emotional and instrumental social support, the entire sample was divided into four subgroups, and three dummy variables were defined, corresponding to groups 2, 3, and 4 (group 1 was considered to be the base group in this study):

Group 1: Low in giving and low in receiving social support

Group 2: Low in giving and high in receiving social support

Group 3: High in giving and low in receiving social support

Group 4: High in giving and high in receiving social support

### 3.4. Ethics Approval

This study was approved by the Ethics Committee of Kochi University of Technology (Application number 156/2018). Written informed consent was obtained from the participants after the objective and purpose of the study were presented on an information sheet.

The questionnaires were collected by the first author, who is a trained psychosocial support facilitator. She received the Community Based Psychosocial Support Facilitators’ Training (CBPSS) from the Nepal Red Cross society. She assured the participants that they could withdraw from the survey at any time if they feel uncomfortable. Consequently, four participants reported feeling uncomfortable and withdrew from the survey. Counseling was offered to those participants immediately after their withdrawal. Counseling includes listening to the problems of the participants related to the earthquake, giving them advice and making them feel comfortable.

## 4. Results

### 4.1. Demographic and Psychological Characteristics of the Sample

The demographic and psychological characteristics of the participants in this study are given in Table 2. The 286 effective responses, from individuals aged 19 to 82 years, with an average age of 44.17 and a standard deviation (SD) of 14.02 years, were recruited for this survey. About 73.4% of respondents were male, and 26.6% were female. About 88.5% were married, and 11.5% were single. The annual average income of the households was 47,360.14 Nepali rupees (NPR). There were 40.2% participants with no education, 36.4% had informal education, and 23.4% had primary education or higher. The average family size was 4.3 individuals. Following the natural disaster, it appeared that 43.4% of the respondents did not have depression, 42.7% had mild depression, 12.6% had moderate depression, and 1.4% had severe depression.

The Cronbach alpha values were 0.73 for giving instrumental support, 0.69 for receiving instrumental support, 0.79 for giving emotional support, 0.82 for receiving emotional support, and 0.63 for the PHQ 9, suggesting that the internal consistency of the measures was acceptable.

### 4.2. Earthquake Exposure Variables

Injuries were suffered by 5.6% of respondents, and 7.3% of respondents reported an injury to a family member. The percentage of the respondents who lost a family member was 8.4%. The houses of 96.5% of respondents were reported to be damaged in the earthquake, and 89.5% lost food stock and reserves. 46.5% lost their livestock. See Table 3 for details.

### 4.3. Preliminary Regression Analysis Result

Before the analysis described in the Materials and Methods section, regression analysis was conducted to interpret the results of the PHQ-9 in terms of the four subscales of social support, assuming that they linearly and independently affected PHQ-9. It was found that the amount of emotional support received was a significant predictor at the 5% level (beta = −0.17, *p* < 0.015). The association fell in a reasonable direction (i.e., more support was associated with lower depression). Regarding instrumental support, the amount of the instrumental support given increased the PHQ-9 scores of individuals at the 1% level (beta = 0.21, *p* < 0.001). See Table 4 for details.

### 4.4. Main Regression Analysis Result

As mentioned in the Materials and Methods section, three dummy variables were defined in relation to the amount of instrumental and emotional support given and received by the respondents, and these were included in the regression model in addition to other covariates. For emotional support, it was found that being high both in giving and receiving support was found to be the only predictor for lower PHQ-9 scores with reference to being low both in giving and receiving at 5% level (beta = −0.17, *p* < 0.024). This suggests an interaction effect of giving and receiving because receiving alleviated depression only if accompanied by giving. With regard to instrumental support, being high in giving and low in receiving increased the PHQ-9 scores for respondents at the 5% level (beta = 0.14, *p* < 0.027), with reference to being low in both giving and receiving. These findings suggest that being high in giving increased the PHQ-9 scores of an individual. See full results in Table 5. Thus, the answers to research questions 1 and 2 were both negative.

## 5. Discussion

This study explored the interaction effects of giving and receiving support on depression severity among earthquake survivors. A quantitative survey was conducted in a rural village of Nepal where external support was limited, and people relied on each other to cope with the effects of the earthquake. This study was guided by the principles of equity theory and norms of reciprocity, which indicate that reciprocal relationships enhance mental health. There were four major findings.

First, when the interaction effects of giving and receiving support were neglected, it was found that the depression level of the survivors decreased with the emotional support received. This indicates the significant beneficial effects of receiving emotional support after a disaster on depression. This result is in line with those of Shakespeare-Finch and Green [17], who reported similar findings in a post-flood scenario in Australia. It seems evident that traumatic events in disaster-affected areas tend to overwhelm the internal resources of individuals (self-esteem, mastery, and purpose in life) [17,61]. Emotional support is effective in such cases, as it provides a strong message of self-worth and competence that can help cope with disaster-induced negative and psychological stress [17,70].

Second, with regard to instrumental support, we found that the depression level of survivors increased when instrumental support was given. This result is in contrast to the findings of Tsuboi et al. [71], Thomas [28] and Momtaz et al. [29], who found that giving instrumental support to others enhanced the psychological well-being of individuals in non-disaster settings. This study was conducted in a disaster setting, and thus the difference may be due to the fact that individuals who experience disasters may face additional psychological challenges or may be in poor health as a result, such that if they provide instrumental support to others in these circumstances, they themselves may experience increased distress. Previous studies have shown that providing instrumental support enhances the psychological well-being of individuals only when they are emotionally engaged in providing instrumental support [26,43,72].

Third, once the interaction effects of giving and receiving social support are taken into account, our findings suggest that receiving additional emotional support alleviates depression only when it is accompanied by giving additional support within a reciprocal relationship. This finding is consistent with that of Maton [40], who found that individuals high in bidirectional support or reciprocity showed the highest level of well-being. Our finding is also consistent with those of Mizuno et al. [47], who found that reciprocal exchange of social support is associated with a low risk of depressive symptoms. This is the first time where equity theory and a theoretical construct of reciprocity have been used to explain the mental health of natural disaster survivors.

An additional aspect should be noted. Strictly speaking, the theories of equity and reciprocity predict that individuals who are high in both giving and receiving emotional support and individuals who are low in both are indifferent to each other with respect to depression severity. However, this study found that the former had better mental health than the latter. This result is clear in relation to the literature of social connectedness and social interaction. In a disaster-affected community, community members may acquire psychological problems and to cope with them, they may participate in mutual helping behavior, which determines the amount of support that they receive and provide [25,71]. In such cases, it seems clear that those with large social networks and strong social connectedness would reciprocate additional support to cope with the negative effects of the disaster they experience [9,14,72,73]. In addition, survivors who participate in mutual exchanges to a greater extent have increased interconnectedness, faith in attitudes toward others, sense of belonging, and social cohesion, all of which decreases depression and increases the individual’s psychological well-being [14,74,75,76,77,78].This suggests that individuals in high reciprocal exchanges have a lower level of depression relative to individuals who have lower levels of reciprocity or non-reciprocal relationships.

Fourth, unlike findings on emotional support, findings regarding instrumental support suggest that net amounts of instrumental support given (rather than the balance between the amount received and that given) matters for the instrumental support, as our statistical results suggest that individuals who are high in giving and low in receiving instrumental support and individuals who are low in both giving and receiving instrumental support are different to each other with respect to the severity of depression. This result seems consistent with Hobfoll’s Conservation of Resources model [79], which posits that a loss of resources (e.g., home, food stocks, mastery, or self-esteem etc.) is related to psychological distress and declining mental health [49,80]. Disaster research shows that survivors are at risk for mental health morbidity, such as anxiety, depression, and psychological stress due to damage to or loss of the resources [80,81]. In these situations, the value of instrumental support for a natural disaster survivor is obvious. However, according to Social Exchange Theory (SET), if the cost of support is higher than the benefits received, individual feel more distressed [82]. Thus, the net amount of support given increased the depression level of individuals. Our findings are also consistent with equity theory, which suggest that people who provide more support than they receive in the reciprocal relationship, such that they may have higher levels of depression than people in other groups.

## 6. Limitations of the Study

This research has some important limitations, which suggest possible directions for future research. First, this research applied a cross-sectional approach. Therefore, future work should investigate the causal effects of giving and receiving support on depression, using a longitudinal study design. Second, we lack pre-earthquake prevalence estimates for depression and estimates of support level from that period, so desirable comparative data is difficult to obtain. Third, the data were collected in a single earthquake-affected village, with a small sample size of 295. Expanding the study to a larger sample and in other disaster-affected areas could develop a clearer picture of mutual helping behavior.

## 7. Conclusions

This study analyzed the impact of reciprocal exchanges of types of social support in the depression status of the survivors in earthquake-damaged community. The results show that the prevalence of depression among the survivors were 42.7 percent with mild depression, 12.6 percent with moderate depression, and 1.4 percent with severe depression. In addition, the results also indicated that amount of emotional support received by the individual alleviated his or her depression only if it was accompanied by giving emotional support. By contrast, the net amount of instrumental support given by the individual increased his or her depression. Overall, this paper demonstrated that reciprocal exchanges of social support are important for minimizing the depression of survivors and to build a disaster-resilient society.

Our results have several practical implications. As far as emotional support is concerned, reciprocity in post disaster recovery is important for maintaining psychological health. In disaster-affected community, where external aid is limited, people are highly dependent on each other for survival. In fact, they share a common vision for how disaster can be tackled to create a resilient community. In such situations, reciprocal exchanges can help create good relationships among individuals and motivate them to cope with the negative effects of disasters. This may reduce the risk of depression as well as other psychological problems among disaster-affected individuals over the long run.

Second, this study found that, so far as instrumental support is concerned, the net amount of instrumental support given in post disaster recovery increases the depression severity of the survivors. In a disaster-affected community, where both the providers and receivers of support are victims, providing more support in this condition makes people more distressed. Therefore, more research is needed to further understand the differences in support imbalances and the costs and benefits of providing support in the context of disaster.

Third, although several years have passed since the earthquake, the effects still persist. Therefore, attention to the affected area should be paid continuously, not only in terms of the provision of the tangible support but also with a focus on the mental health of the survivors. Furthermore, local authorities should take the initiative to identify pre-existing support (youth clubs, women groups, and religious institution) and strengthen them to improve their cohesion to minimize mental health problems. Humanistic concerns such as early identification of cases, ongoing monitoring, and sustained psychosocial support should be offered.

## Figures and Tables

**Table 1:** Measures of 2-way social support.

**Giving emotional support**
1. Did people confide in you when they had problems? *
2. Did you look for the ways to cheer people when they were feeling low and down during and after earthquake? *
3. Did you provide a sense of comfort to others during and after the earthquake? *
4. Did you provide help to others by listening to their earthquake-induced problems? *
5. Did people close to you share their fear and worries caused by the earthquake? *
**Receiving emotional support**
6. Did you share your fear and anxiety caused by the earthquake with others? *
7. After the earthquake, did you feel that there was someone whom you could trust? *
8. Did you share your thoughts with someone close to you when you felt low or down after the earthquake?*
9. Do you have someone who makes your life feel worthwhile after the earthquake? *
10. After the earthquake, did you feel that you had a circle of people who valued you? *
11. Did you tell someone close to you about the problems you had caused by earthquake? *
**Giving instrumental support**
12. Did you provide rescue support to those who were injured after the earthquake? ***
13. After the earthquake, did you help to dig victims out of damaged houses? **
14. After the earthquake, did you help an injured person seek medical attention? ***
15. After the earthquake, did you provide support to others to dig out their food and clothes from their damaged home? **
**Receiving instrumental support**
16. Did you receive rescue support from others following earthquake to save your injured family members? ***
17. Did you receive support from others to seek medical attention when you or your family member was injured after the earthquake? ***
18. Did you receive support from others to dig victims out of your damaged house after the earthquake? **
19. Did you receive support from others to dig out your food and clothes from your damaged home? **

Note: * items adopted from Shakespeare-Finch and Obst (2011); ** items adopted from Bokszczanin (2011); *** items created by authors.

**Table 2:** Demographic and psychological characteristics of the sample (*n* = 286).

Variables	*n*	%	*M*	*SD*	*Cronbach’s Alpha*
**Gender**					
Male	210	73.4%			
Female	76	26.6%			
**Age**			44.17	14.02	
**Marital status**					
Married	253	88.5%			
Single	33	11.5%			
Household income (NPR)			47,360.14		
**Education status**					
No education	115	40.2%			
Informal education	104	36.4%			
Primary education or above	67	23.4%			
Family size			4.3		
**Severity of depression**					
None (0–4)	124	43.4%			
Mild depression (5–9)	122	42. 7%			
Moderate depression (10–14)	36	12.6%			
Severe depression (≥15)	4	1.4%			
**Social support scale**					
Giving instrumental support (4 items)					0.73
Receiving instrumental support (4 items)					0.68
Giving emotional support (5 items)					0.79
Receiving emotional support (6 items)					0.82
Patient Health Questionnaire-9 (PHQ-9)					0.63

**Table 3:** Earthquake exposure variables (*n* = 286).

Variables	Frequency	Percentage (%)
Suffered injury	16	5.6
Injured family members	22	7.3
Loss of family members	24	8.4
Damaged home	276	96.5
Loss of livestock	133	46.5
Loss of food stock	256	89.5

**Table 4:** Linear regression analysis predicting depression severity.

Variables	β		*p*-Value	Std. err.
Giving instrumental support	0.21	***	0.001	0.07
Receiving instrumental support	−0.09		0.264	0.07
Giving emotional support	0.00		0.913	0.07
Receiving emotional support	−0.17	**	0.015	0.07
**Sociodemographic variables**				
Age	0.06		0.352	0.07
Sex	−0.11		0.092	0.06
Household Income ^1^	0.07		0.272	0.07
Married	−0.11		0.071	0.06
Family size	−0.004		0.945	0.07
**Education status**				
No education (base group)				
Informal education	0.02		0.763	0.07
Primary education or above	−0.09		0.192	0.08
**Earthquake exposure variables**				
Suffered injury	−0.06		0.337	0.07
Injured family members	0.00		0.998	0.06
Loss of family members	0.08		0.175	0.06
Damaged home	−0.03		0.652	0.06
Loss of food stock	0.06		0.333	0.06
Loss of livestock	−0.11		0.055	0.06
Total observations	286			
R-squared = 0.12; Adj. R-squared = 0.07				

Notes: *** *p* < 0.01, ** *p* < 0.05. No education is taken as the base group for education. ^1^ Regression analysis are computed with the natural logarithm of annual household income.

**Table 5:** Interaction effects of social support on depression severity.

Variables	β		*p*-Value	Std. err.
**Emotional support**				
Low giving—low receiving (base group)				
Low giving—high receiving	−0.02		0.707	0.06
High giving- low receiving	0.10		0.129	0.06
High giving—high receiving	−0.17	**	0.024	0.08
**Instrumental support**				
Low giving—low receiving (base group)				
Low giving—high receiving	−0.14		0.065	0.07
High giving—low receiving	0.14	**	0.027	0.06
High giving—high receiving	0.09		0.178	0.07
**Sociodemographic variables**				
Age	0.10		0.203	0.07
Sex	−0.10		0.126	0.06
Household Income ^1^	0.08		0.237	0.07
Married	−0.11		0.070	0.06
Family size	−0.07		0.323	0.07
Number of children	0.11		0.167	0.07
**Education status**				
No education (base group)				
Informal education	0.02		0.759	0.06
Primary education or above	−0.09		0.227	0.07
**Earthquake exposure variables**				
Suffered injury	−0.03		0.656	0.07
Injured family members	−0.03		0.653	0.07
Loss of family members	0.09		0.124	0.06
Damage home	−0.02		0.702	0.06
Loss of food stock	0.08		0.229	0.06
Loss of livestock	−0.09		0.096	0.05
Total observations	286			
R-squared = 0.15; Adj. R- squared = 0.09				

Notes: ** *p* < 0.05, No education is taken as the base group for education. ^1^ Regression analysis are computed with the natural logarithm of annual household income.

## Data Availability

Not applicable.

## Appendix A

▪ 2-Way Social Support Measures
▪ The following items were not included in the final analysis (*** created by authors):

**A. Providing social support (5 items)**
1. Did you provide space for others to build temporary housing in your barren land? ***
2. Did you provide space for others to build their temporary housing in your farmland? ***
3. Did you provide monetary assistance to someone in the village while they faced with any economic challenges during and after earthquake without charging any interest rate? ***
4. Did you give financial assistance to someone while they face with any economic challenges during and after the earthquake with charging the interest rate? ***
5. Did you provide financial support to the injured person/family to seek medical treatment? ***
**B. Receiving social support (4 items)**
1. Did anyone support you by providing space of their land for your temporarily settlement after the earthquake? ***
2. After the earthquake, did you receive temporary shelter assistance from others? ***
3. Did you receive monetary assistance from others while faced with any economic challenges during and after the earthquake? (without being charged of interest rate) ***
4. Did you receive monetary assistance from others while faced with any economic challenges during and after the earthquake? (With being charged of interest rate)? ***
